# Cognitive and behavioral functioning in two neurogenetic disorders; how different are these aspects in Duchenne muscular dystrophy and Neurofibromatosis type 1?

**DOI:** 10.1371/journal.pone.0275803

**Published:** 2022-10-10

**Authors:** Danique M. J. Hellebrekers, Sandra A. M. van Abeelen, Coriene E. Catsman, Sander M. J. van Kuijk, Annick M. Laridon, Sylvia Klinkenberg, Jos G. M. Hendriksen, Johan S. H. Vles

**Affiliations:** 1 Centre for Neurological Learning Disabilities, Kempenhaege, Heeze, The Netherlands; 2 School for Mental Health and Neuroscience, Maastricht University, Maastricht, The Netherlands; 3 Department of Neurology, Erasmus Medisch Centrum, Rotterdam, The Netherlands; 4 Department of Clinical Epidemiology and Medical Technology Assessment, Maastricht University Medical Centre, Maastricht, The Netherlands; 5 Department of Neurology, Maastricht University Medical Centre, Maastricht, The Netherlands; 6 Duchenne Centre Netherlands, Nijmegen and Leiden, The Netherlands; University of Minnesota Medical School, UNITED STATES

## Abstract

The presence of neurocognitive and behavioral problems are common features in various neurogenetic disorders. In Duchenne muscular dystrophy (DMD), these problems have been linked to mutations along the dystrophin gene affecting different brain dystrophin isoforms. However, comparable cognitive and behavioral problems have been found in Neurofibromatosis type 1 (NF1). This study aims to assess disorder specific differences in cognition and behavior between DMD and NF1. Retrospective data of 38 male patients with DMD were aged-matched with data of 38 male patients with NF1. Patients of both groups underwent neurocognitive assessment for regular clinical care. Intellectual abilities, sequential and simultaneous processing, verbal memory and sustained attention were evaluated. In addition, parents and teachers completed behavioral questionnaires. Males with DMD exhibited low intellectual abilities and sequential processing problems, but these outcomes not significantly differed from males with NF1. Simultaneous processing, verbal memory and sustained attention outcomes were equal for both groups. Outcomes of questionnaires displayed higher rates of aggressive behavior (13.2%) in DMD, whereas in NF1 higher rates of problems with thinking (15.8%), withdrawn (10.5%) and social behavior (10.5%) were noticed. In the neurogenetic disorders DMD and NF1, on average overlapping cognitive and behavioral problems are noticed, suggesting that these are not only caused by gene mutations resulting in a lack of one specific protein.

## Introduction

There is growing evidence that gene mutations can cause abnormal brain development that lead to cognitive and behavioral problems in patients with neurogenetic disorders such as Duchenne muscular dystrophy (DMD), Neurofibromatosis type 1, 22q11.2 deletion syndrome, Prader-Willi syndrome, fragile X syndrome and Turner syndrome [1–6]. In DMD, gene mutations result in a loss of the full-length dystrophin protein isoform (*Dp427*) in muscles (M) and the brain (B) [7, 8]. A lack of the dystrophin protein *Dp427*_*M*_ is responsible for the progressive muscle weakness in DMD [9]. The isoform *Dp427*_*B*_ and three shorter brain isoforms *Dp140*, *Dp71+Dp40* are believed to be expressed throughout the cerebral cortex with the highest expression in the temporal and frontal cortex, the amygdala and hippocampus [10–13]. The production of one of the brain isoforms (*Dp140*) is particularly elevated during fetal life stages, suggesting that it may influence brain development [11].

Patients with Duchenne frequently exhibit cognitive problems, neurodevelopmental-, and behavioral disorders [3, 10, 14, 15]. The full-scale intelligence quotient (FSIQ) in DMD is on average one standard deviation below the population mean [16]. In addition, problems with verbal working memory, attention, executive functioning, learning (e.g. reading, writing, math) have been reported [14, 16–18]. The higher rates of neurodevelopmental and behavioral disorders are found for attention-deficit hyperactivity disorders (ADHD; up to 32%), autism spectrum disorders (ASD; up to 21%), obsessive compulsive disorders (OCD up to 5.1%) and anxiety (up to 27%), the numbers vary due to use of various (screening) instruments [14, 19–22]. Recent studies have tried to assess whether specific gene mutations that affect the production of brain isoforms can be related to the cognitive problems, neurodevelopmental-, and behavioral disorders of patients with DMD [10, 11, 14, 21–24]. It seems that patients with mutations affecting multiple brain isoforms exhibit more severe problems in cognition and behavior than patients missing only *Dp427*_*B*_ [19, 21, 23–26].

Neurofibromatosis type 1 (NF1) is caused by germline mutations in the NF1 gene, resulting in a decreased production of the tumor suppressive protein, neurofibromin [27].

There are a broad number of possible mutations in the large NF1 gene, resulting in variable phenotypes with various neurocutaneous manifestations including (plexiform) neurofibromas, café-au-lait spots, skinfold freckling, but also skeletal and muscular problems (e.g. scoliosis, pseudo-arthrosis, decreased bone strength, reduced muscle strength and motor problems) [27, 28]. Previous studies in mice have showed that deletions involving exons *NF1-23a* and *NF1-9a* result in altered isoform expression in the brain i.e. in astrocytes and in neurons of the striatum, cortex and hippocampus [29]. Due to the role of neurofibromin in the brain, human and mice studies have linked a lack of this protein to the cognitive and learning disabilities that are found for NF1 [29–32]. Low-average IQ levels are usually shown in patients with NF1, but impairments are also found in visuo-spatial perceptual and visuomotor skills, language, learning (e.g. reading) and executive functions (e.g. attention and working memory) [31–35]. In addition, in NF1 higher prevalence rates of ADHD (up to 50%), ASD (14%) and behavioral problems such as anxiety and depression (43%), have been noticed compared to the general population [31–33, 35–40]. Due to the large number of unique mutations in NF1, it is complicated to define a distinct cognitive and behavioral profile [31]. However, recent NF1 studies have found distinct profiles and showed that patients with microdeletions display more pronounced cognitive impairments and learning disabilities than patients with intragenic mutations [31, 41–43].

For neuropsychological diagnostic work-up and treatment purposes we were interested whether patients with different neurogenetic disorders such as DMD and NF1 have specific cognitive and behavioral profiles. In both disorders it has frequently been shown that specific cognitive and behavioral comorbidities occur [3, 10, 14, 15, 29–33, 35–40]. Current literature on DMD and NF1 shows that the presence of these problems may be correlated to specific genetic mutations i.e. in DMD this concerns mutations affecting multiple brain isoforms [19, 21, 23–26] and in NF1 this concerns having microdeletions [31, 41–43]. We were interested whether the cognitive and behavioral profiles of these two neurogenetic disorder can be distinguished to assume that different genetic mutations affecting different proteins indeed cause specific profiles. Therefore, the current study aimed to assess whether the cognitive and behavioral impairments differ between DMD and NF1.

## Materials and methods

### Study population

Eligible patients for current study were males with DMD and males with NF1 attending to the outpatient clinic of Kempenhaeghe, the Centre for Neurological Learning Disabilities (CNL), Heeze, the Netherlands, as this Centre is predominantly responsible for the neuropsychological care of these patients in the Netherlands. The inclusion criteria comprised of (1) having a previous genetically confirmed mutation of the dystrophin gene for patients with DMD, or (2) having a clinical diagnosis of Neurofibromatosis type 1 or a previous genetically confirmed mutation of the neurofibromin gene, (3) an age between 6–16 years, (4) an adequate proficiency in Dutch, (5) normal hearing, (6) absence of severe visual impairment and (7) no physical immobility of upper extremities (the reliability of the cognitive tests may be impaired by impairments in hearing, vision and physical immobility of upper extremities). The previously genetic confirmed mutations of DMD and NF1 were established by medical professionals according to the specified criteria [44–46]. Exclusion criteria were: epilepsy, symptomatic optic pathway glioma, brain tumors, hydrocephalus or brain abnormalities (e.g. cortical dysplasia). Males with NF1 with focal abnormal signal intensity were not excluded because no equivocal relation is assumed between the presence of focal abnormal signal intensity and cognitive, developmental impairments and learning disabilities [47, 48]. Each eligible male patient with DMD was matched on age (restriction within 1 year) to an age equivalent male with NF1. The age range of participants (6–16 years) was chosen to allow for the administration of the cognitive test and behavioral questionnaires, standardized for the Dutch population. All patients of CNL give at the beginning of their regular care process verbally consent to use their data for scientific purposes. Since the start of the new European law (5^th^ Mai 2018) concerning using personal data 2, patients had to give written consent. Thereby patients included in current study give their verbal or written consent, which is documented in the patient file. Ethical approval was granted by the local Medical Ethical Committee of Kempenhaeghe. The study was conducted in accordance with the 18^th^ World Medical Assembly, Helsinki 1994.

### Study procedure

All patients with DMD and NF1 received an extensive neuropsychological assessment between October 2008 and August 2019 to evaluate their cognitive and behavioral functioning as part of regular clinical care at CNL. Cognitive assessment evaluated intellectual abilities (FSIQ, verbal intelligence and performance intelligence), processing speed, sequential processing (verbal span capacity and working memory), simultaneous processing (visuospatial functioning), verbal memory (immediate recall, delayed recall, recognition) and sustained visual- and auditory attention. Behavioral functioning was screened using questionnaires for parents and teachers. All collected cognitive and behavioral data were extracted from the patient files for current retrospective study. Demographic (i.e. age, educational level, gender), disease-related characteristics (i.e. genetic mutation, ambulation, comorbid learning disabilities, neurodevelopmental or behavioral Diagnostic and Statistical Manual (DSM) classified diagnoses, use of stimulant medication such as methylphenidate (MPH), use of corticosteroids, somatic comorbidities, vision or hearing problems and immobility of upper extremities), sociodemographic characteristics of parents and information on problems during pregnancy and delivery were extracted from the patient files. The comorbid learning disabilities extracted from the patient files included dyslexia and dyscalculia. Learning difficulties such as problems with reading, writing, math, automatization or spelling that did not fulfill the criteria for dyslexia and dyscalculia were extracted from the files. The neurodevelopmental and behavioral DSM-IV/DSM-5 that were obtained from the patient files included ADHD, ASD, OCD, developmental coordination disorder, anxiety, depression and tic disorders. All cognitive, behavioral and learning comorbidities were previously diagnosed by a health or medical professional. The educational status of patients was categorized as regular or special education. Parents educational status was indicated using the Dutch Verhage categories [49] and was used to estimate the sociodemographic status of patients. The Verhage categories were combined into (1) low level (i.e. <6 years of primary education, finished primary education, <2 years low-level secondary education, finished low-level secondary education), (2) middle level (i.e. finished average-level secondary education) and (3) high level (i.e. finished high level secondary education, university degree) [49].

### Neuropsychological assessment

#### Cognition

The Wechsler Intelligence Scale for Children-Third edition (WISC-III) [50] measured Full-Scale Intelligence Quotient (FSIQ), Verbal Intelligence (VIQ), Performance Intelligence (PIQ), Verbal Comprehension, Perceptual Organization and Processing Speed. Raw scores of the WISC-III were converted to age-related norm scores (mean = 100, SD = 15) [50]. The Kaufmann Assessment Battery for Children-II (KABC-II) was used to assess sequential processing (verbal span and auditory working memory) and simultaneous processing (visuospatial functioning) [51]. Sequential processing was based on the subtests Number recall and Word Order. Simultaneous processing was based on the subtests Rover and depending on age the subtests Triangles (6 years) or Block Counting (7–16 years). Raw scores of the subtests were converted to age-related scaled scores (mean = 100, SD = 15) [51]. Verbal memory of immediate recall, delayed recall and recognition was tested using the Rey auditory learning task (15-word test) [52]. Scores of the 15-word test were computed to (1) a sum of correct responses given during the five consecutive trials (total immediate recall score), (2) total correct response during the delayed trial (delayed recall score) and (3) sum of correct recognition responses (recognition score) [52]. Sustained visual attention was measured using the Bourdon Vos [53]. The Test of Everyday Attention for Children, Second Edition (TEA-Ch) [54], subtest Score was used to measure sustained auditory attention. Teach-Ch raw scores were converted to scaled scores (mean = 10, SD = 3) [54]. In our Centre, the outcomes of WISC-III and KABC-II are both evaluated as WISC tasks involving time pressure may negatively influence outcomes of DMD patients due to less functioning of upper extremities.

#### Behavior

Behavioral functioning was screened using two informant rating instruments, the Child Behavior Checklist for Children (CBCL) and the Teacher report Form (TRF) [55]. Both instruments evaluated behavior based on eight syndrome scales (anxious/depressed, withdrawn/depressed, somatic complaints, social problems, thought problems, attention problems, rule-breaking behavior and aggressive behavior). Two broadband scales on internalizing symptoms (made up of withdrawn, somatic complaints and anxious/depressed scales), externalizing symptoms (made up of rule-breaking behavior and aggressive behavior), and a total problem scale score were calculated using the syndrome scale scores. In line with the manual, a cut-off value (clinical range score) of T≥70 was used to indicate the clinical range of the eight syndrome scales, and T≥64 was applied to indicate the clinical range of internalizing, externalizing symptoms and a total problem score [55].

### Statistical analysis

Age-matching (restriction within 1 year) was randomly performed by case control matching of SPSS. Demographic and disease-related characteristics of both groups were presented as mean (SD), or absolute number and proportion. Stochastic regression imputation was applied in case of incomplete variables of cognitive and behavioral data [56]. The imputed values were drawn using predictive mean matching [56]. Differences between the DMD and NF1 group on demographic and disease-related parameters as well as cognitive and behavioral outcomes were tested using the independent samples t-test, X^2^ tests, Fisher exact test, or Mann-Whitney-U tests, as appropriate. Differences within the DMD and NF1 group on the cognitive outcomes of KABC sequential and simultaneous processing were tested using paired samples t-test. Effect sizes (quantified as Cohen’s d) were calculated to indicate the strength of differences of the cognitive and behavioral outcomes [57]. Effect sizes were defined as: 0.20–0.50 = small, 0.50–0.80 = medium and ≥0.80 = large [57]. Multivariate analyses (MANOVA) examined differences between the groups on cognitive and behavioral outcomes corrected for the covariates age, comorbid diagnoses of patients (i.e. ADHD and ASD), use of stimulant medication (MPH), educational status of patients and family history of learning and behavioral problems. Preliminary assumptions associated with all test statistics, such a normality and multivariate normality, homogeneity of variance, homogeneity of variance-covariance matrices, linearity and multicollinearity were examined using a variety of methods including visual inspection of histograms, boxplots, scatterplots, inspection of skewness, kurtosis, Shapiro-Wilk test, Levene’s test and Box’s M test (*p* ≥ .001) [58]. Cognitive outcomes i.e. age-related norm scores were converted to z-scores (Mean = 0, SD = 1). Behavioral outcomes were also evaluated using the clinical cut-off value of T-score ≥63 [52]. All statistical analyses were carried out using IBM SPSS version 24.0 for MAC OS X.

## Results

### Participant characteristics

**Fig 1:**
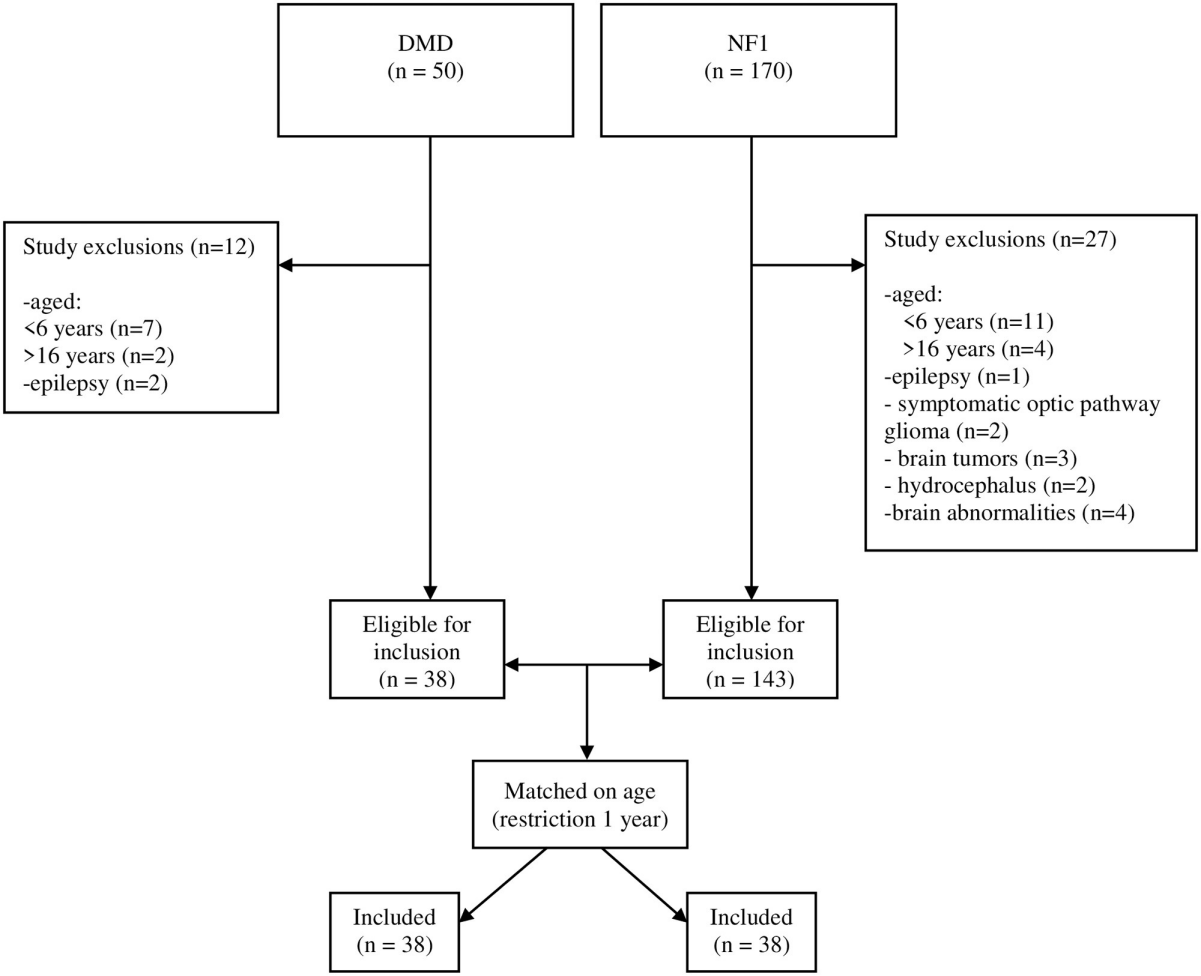
Flowchart of inclusion. Note: DMD = Duchenne muscular dystrophy, NF1 = Neurofibromatosis type 1.

Data of 50 patients with DMD of 170 patients with NF1 were available (Fig 1 for flowchart of inclusion). A total of 38 patients with DMD were matched on age with males with 38 NF1. Demographic and disease-related characteristics of both groups are displayed in Table 1.

**Table 1:** Participant characteristics.

	DMD (N = 38)	NF1 (N = 38)	*p*
**Demographic characteristics**			
Mean age in years (SD)	9.6 (2.6)	9.7 (2.6)	.839
Education of patients (%)			.000**
Regular education	6 (15.8)	23 (60.5)	
Special education	32 (84.2)	15 (39.4)	
Educational levels of parents (%)			
Mother:			.522
Low level	6 (17.1)	4 (10.5)	
Middle level	14 (40.0)	17 (56.7)	
High level	15 (42.9)	9 (30.0)	
Father:			.570
Low level	4 (10.5)	2 (7.1)	
Middle level	6 (19.4)	10 (35.7)	
High level	21 (67.7)	16 (57.1)	
Family history learning and behavioral problems (%)			.002*
ADHD	4 (10.8)	9 (24.3)	
ASS	1 (2.7)	4 (10.8)	
Dyslexia	6 (16.2)	13 (35.1)	
Learning difficulties	3 (8.1)	3 (8.1)	
Pregnancy & delivery problems (%)			.229
Hypoxia	1 (2.6)	0	
Premature birth (34 to ≤ 37 wk)	4 (10.4)	1 (2.6)	
C-section	1 (2.5)	2 (5.3)	
Intrauterine growth problems	2 (5.3)	2 (5.3)	
Pre-eclampsia	1 (2.6)	0	
**Disease-related characteristics**			
Wheelchair dependence (%)			.000**
Permanent	16 (44.4)	0	
Intermittent	5 (13.9)	0	
Never	15 (41.7)	0	
Medication use (%)			
Steroids (prednisone)	31 (81.6)	0	
Stimulants (MPH)	4 (10.5)	9 (23.7)	.222
Sleep problems (%)			.133
Falling asleep	8 (21.6)	15 (39.5)	
Staying asleep	0	0	
Comorbid diagnoses (%)			.491
ADHD	7 (18.2)	16 (41.6)	.025*
ADD	3 (7.9)	4 (10.5)	1.000
ASD	10 (26.3)	4 (10.5)	.076
Depression	1 (2.6)	0	1.000
Anxiety	2 (5.3)	0	.493
Tics	1 (2.6)	1 (2.6)	1.000
Dyslexia	1 (2.6)	4 (10.5)	.358
Dyscalculia	3 (7.9)	0	.240

Note: Results are mean (SD) or median (range) for continuous variables, and frequencies (%) for categorical variables. Verhage categories are defined as low level (i.e. <6 years of primary education, finished primary education, <2 years low-level secondary education, finished low-level secondary education), middle level (i.e. finished average-level secondary education), and high level (i.e. finished high level secondary education, university degree).^41^ wk = weeks, AD(H) D = Attention-deficit (hyperactivity disorder), ASD = Autism Spectrum Disorder. Reasons for C-section were: N = 1 pelvic presentation, N = 1 C-section at 38 weeks because of intrauterine growth problems, and N = 1 emergency C-section but reason was not documented. Reports on family history of learning and behavioral problems are based on 1^st^, 2^nd^, and 3^rd^ family degree.

* p < .05 (two-sided)

** p < .01 (two-sided)

Of the DMD group, 21 males (55.3%) had mutations affecting *Dp140* production (i.e. mutations corrupting the *Dp140* promoter, the *Dp140* translation start site or located downstream of exon 50 as the *Dp140* ATG start-site is located in exon 51). Ten males (26.3%) had mutations not affecting *Dp140* production (i.e. deletions or duplications upstream of intron 44). Dystrophin expression was undefinable of five males (13.2%) with deletions or duplication breakpoints between intron 44 and exon 51.^24^ No information on deletions or duplications was available in the electronic patient files of two males (5.3%). Neurofibromatosis type 1 was clinically diagnosed in N = 38 males and of n = 32 information on mutation location was available. In n = 4 the clinical diagnosis is not genetically confirmed and of n = 2 information of mutation location is missing. None of the 32 patients of which mutation information was available had microdeletions (see S1 Table).

Disease-related characteristics are displayed in Table 1. The majority of the DMD group (81.6%) used prednisone steroids, six (15.6%) used deflazacort and one (2.6%) had no corticosteroid treatment, because it severely affected his emotional status. The prevalence rates of comorbid diagnoses in neurodevelopmental and behavioral disorders differed between the DMD and NF1 group (see Table 1). ASD diagnoses were more often found for the DMD group (23.4%) compared to the NF1 group (13%), whereas the rate of ADHD diagnoses is higher for the NF1 group (41.6%) than for the DMD group (18.2%, see Table 1). The difference in rate of ADHD between the groups is even statistically significant (Table 1). Diagnoses of learning disorders such as dyslexia and dyscalculia were found in both groups. Furthermore, n = 11 males with DMD (28.6%) and n = 18 (46.8%) males with NF1 exhibited learning disabilities in reading, writing, mathematics, spelling and automatization that not fulfil the diagnostic criteria for dyslexia or dyscalculia. Within the NF1 group nine used MPH, whereas in the DMD group four males used MPH (see Table 1).

### Cognitive outcomes

#### Intellectual abilities

As shown in Table 2, no discrepancy was found between VIQ and PIQ for the DMD and NF1 group. On all IQ measures no significant differences were found between the DMD and NF1 group.

**Table 2:** Wechsler Intelligence Scale for Children-III outcomes of the DMD and the NF1 group.

	Mean (SD) DMD group (N = 38)	Mean (SD) NF1 group (N = 38)	Test-statistic value	*p*	*Effect size*	95% CI	
						Lower	Upper
FSIQ	86.4 (11.9)	91.5 (15.4)	-1.626	.108	-0.4	-11.42	1.16
VIQ	89.6 (12.0)	93.9 (14.4)	-1.387	.170	-0.3	-10.32	1.85
PIQ	85.3 (12.0)	89.9 (17.0)	-1.370	.175	-0.3	-11.38	2.11
VC	91.5 (9.5)	95.5 (15.0)	-1.411	.163	-0.3	- 9.81	1.69
PO	84.9 (8.6)	89.4 (14.5)	-1.654	.103	-0.4	-9.98	0.95
PS	89.5 (16.2)	94.0 (17.3)	-1.402	.165	-0.3	-12.17	3.15

Note: Mean (SD) of scaled scores, Test statistic values are t-values, Effect size = Cohen’s d, 95% CI = 95% Confidence Interval. FSIQ = Full-scale intelligence quotient, VIQ = Verbal intelligence quotient, PIQ = Performance intelligence quotient, VC = Verbal Comprehension, PO = Perceptual Organization, PS = Processing speed

**Fig 2:**
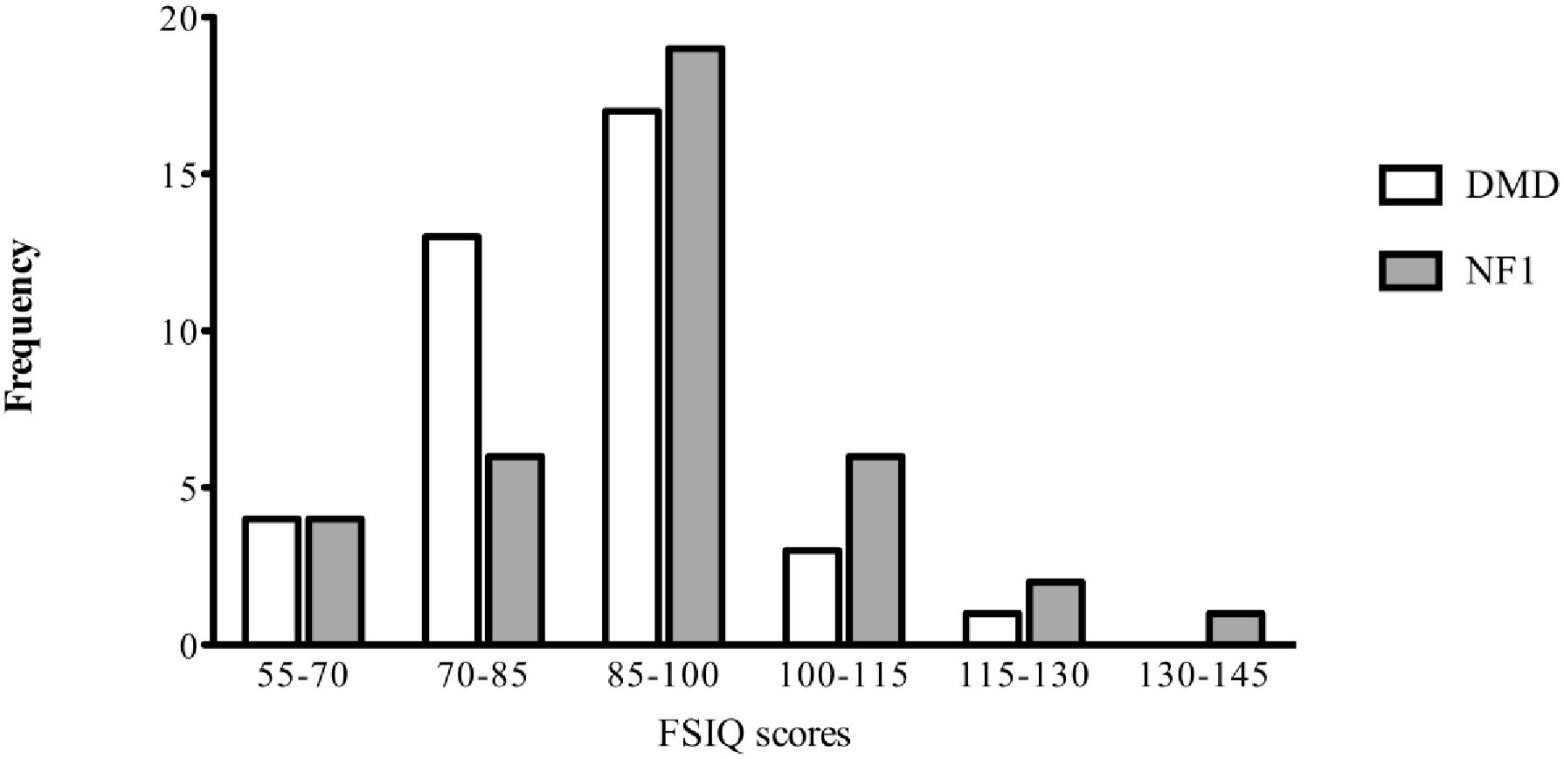
Frequencies of the Wechsler full-scale intelligence quotient scores of the DMD (N = 38) and NF1 group (n = 38). FSIQ = full-scale intelligence quotient, DMD = Duchenne muscular dystrophy, NF1 = Neurofibromatosis type 1. FSIQ mean scores are displayed using frequencies of the group DMD (white) and group NF1 (grey) patients.

WISC distribution of FSIQ of the two groups are displayed in Fig 2. Of the DMD group, four males (10.5%) had a FSIQ score of ≤70, thirteen males (34.2%) scored between 70–85, seventeen (44.7%) fell within the range 85–100, three (7.9%) scored between 100–115 and one (2.6%) had a FSIQ score ≥115 (Fig 2). Of the NF1 group, four (10.5%) scored below ≤70, six males (15.8%) had a FSIQ score between 70–85, nineteen (50.0%) fell within the range 85–100, six (15.8%) had a FSIQ score between 100–115 and three (7.9%) had FSIQ score ≥115 (Fig 2). The sociodemographic status (measured by educational status (ES) of parents) of both groups were not correlated to the FSIQ outcomes (ES mothers of DMD group *rs =* .16, *p* >.05, ES fathers of DMD group *rs =* .19, *p* >.05, ES mothers NF1 group, *rs =* .13, *p* >.05 and ES fathers NF1 group, *rs =* .26, *p* >.05).

#### Sequential and simultaneous processing

Results of mean sequential processing and mean simultaneous processing of the two groups are displayed in Table 3. No significant difference was found between the groups on sequential processing and simultaneous processing (see Table 3).

**Table 3:** Cognitive outcomes of (working) memory, attention, and visuospatial abilities of the DMD and NF1 group.

*Cognitive domains*	DMD (N = 38)	NF1 (N = 38)	Test-statistic value	*p*	*Effect size*	95% CI	
						Lower	Upper
*SEQ*	-1.21 (0.84)	-1.29 (0.53)	0.536	.594	0.1	-0.23	0.41
*SIM*	-0.30 (0.96)	-0.17 (0.80)	-0.620	.537	-0.1	-0.53	0.28
*SVAS*	-0.55 (1.01)	-0.70 (1.04)	0.619	.538	0.1	-0.32	0.61
*SVAA*	-0.65 (1.27)	-0.51 (1.36)	-0.468	.641	-0.1	-0.74	0.46
*SAU*	-0.66 (1.08)	-0.85 (1.05)	0.767	.445	0.2	-0.30	0.67
*IR*	-0.27 (1.17)	-0.24 (1.64)	-0.075	.941	-0.0	-0.67	0.63
*DR*	-0.38 (1.14)	-0.54 (1.35)	0.542	.589	0.1	-0.42	0.73
RC*	28.5 (24–30)	29 (21–30)	-0.890	.374	NA	NA	NA

Note: Z-scores are mean (SD) except of * outcomes are raw median (range) scores, Test-statistic values are t-values, except of * is z-value, Effect size = Cohen’s d, 95% CI = 95% Confidence Interval. SEQ = Sequential processing, SIM = Simultaneous processing, SVAS = Sustained Visual Attention Speed, SVAA = Sustained Visual Attention Accuracy, SAU = Sustained Auditory Attention, IR = Immediate Recall, DR = Delayed Recall, RC = Recognition, DMD = Duchenne muscular dystrophy, NF1 = Neurofibromatosis type 1, NA = not applicable.

**Fig 3:**
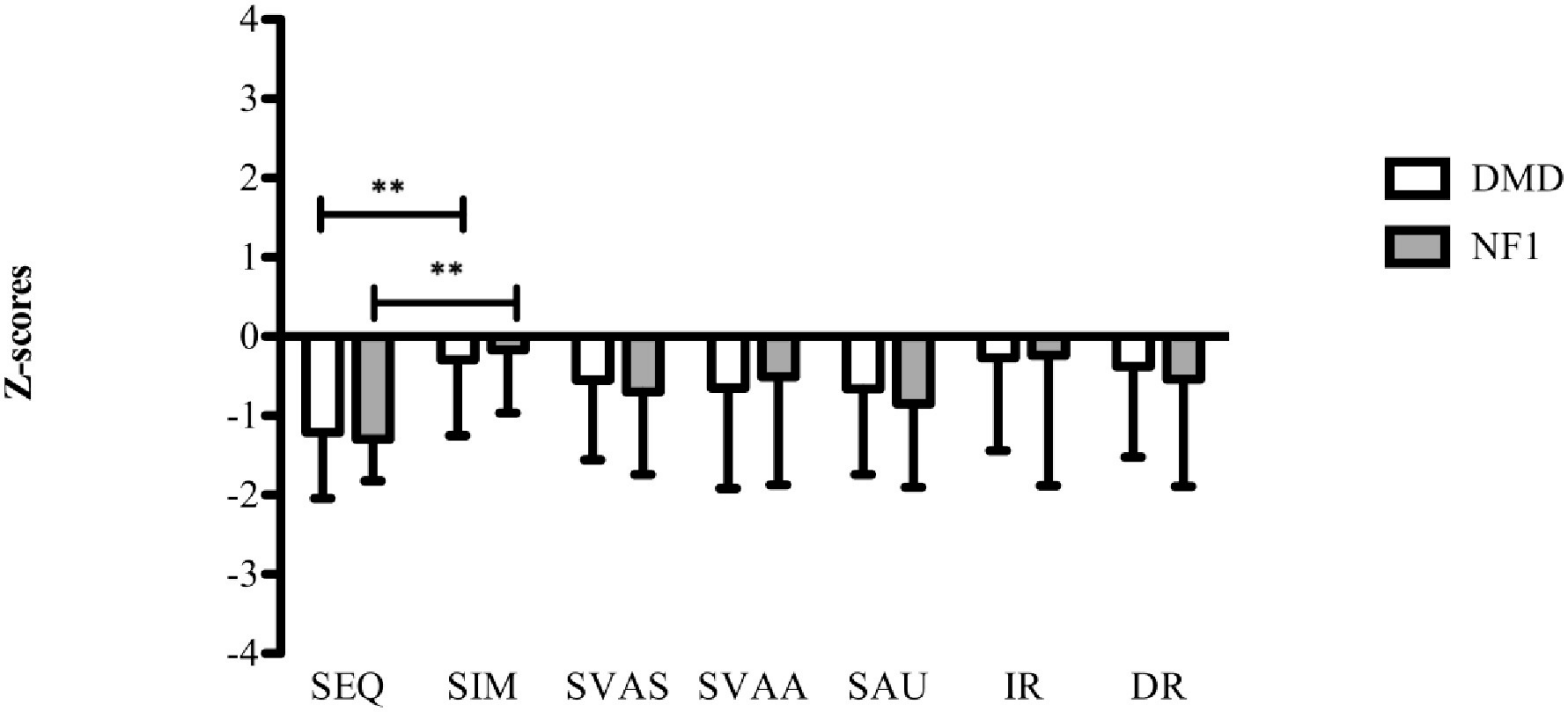
Mean (SD) outcomes of z-scores of the DMD group (N = 38) and NF1 (N = 38) group. SEQ = Sequential processing, SIM = Simultaneous processing, SVAS = Sustained Visual Attention Speed, SVAA = Sustained Visual Attention Accuracy, SAU = Sustained Auditory Attention, IR = immediate recall, DR = delayed recall, DMD = Duchenne muscular dystrophy, NF1 = Neurofibromatosis type 1. ** p < .001 (two-sided). The outcomes are frequencies of the mean outcomes. The statistical method used to compare the outcomes of KABC SEQ processing and SIM processing within the DMD or NF1 group was the paired sample t-test. The z-scores represent the mean outcomes and standard deviations on cognitive outcomes of each group.

See Fig 3 for visualization of differences between the DMD and NF1 group on outcomes of sequential and simultaneous processing. Both groups had lower sequential processing than simultaneous processing outcomes (DMD group, *p* < .001 and NF1 group, *p* < .001). No significant correlation was found between the lower sequential outcomes and FSIQ outcomes of the DMD population (*r =* 0.23, *p* >.05) and NF1 population (*r =* 0.05, *p* >.05). Simultaneous processing outcomes were moderate but significantly correlated with FSIQ in the DMD group (*r =* 0.39, *p* < .05), but not in the NF1 group (*r =* 0.08, *p* >.05).

#### Verbal memory

On verbal memory i.e. immediate recall, delayed recall and recognition no significant differences were found between the groups (see Table 3). The outcomes on immediate and delayed recall of both groups are visualized in Fig 3. Only immediate recall of the NF1 group was significantly correlated with total FSIQ (*r =* 0.55, *p* < .001) and no correlation was found for the DMD group (*r =* 0.06, *p* >.05).

#### Sustained attention

Results on the sustained attention tasks (i.e. visual speed and accuracy as well as auditory attention) showed no significant differences between the DMD and NF1 group (see Table 3). See Fig 3 for visualization of differences between the DMD and NF1 group on outcomes of sustained visual and auditory attention. All sustained attention measures were not significantly correlated with the FSIQ outcomes of the DMD and NF1 group.

A multivariate analysis was run to determine the effect of the covariates, age, comorbid diagnoses of patients (i.e. ADHD and ASD), use of stimulant medication (MPH), educational status of patients and family history of learning and behavioral problems on all cognitive outcomes of both groups. Results showed again non-significant differences between the groups on intellectual abilities, sequential and simultaneous processing, verbal memory, sustained visual and auditory attention after controlling for the influence of the covariates on the cognitive outcomes (see Table 4).

**Table 4:** Multivariate analyses of cognitive outcomes of the DMD and NF1 group.

	DMD (n = 38)			NF1 (n = 38)			*p*
	Mean	95% CI		Mean	95% CI		
		Lower	Upper		Lower	Upper	
Cognitive outcomes							
*FSIQ*	-0.77	-1.10	-0.44	-0.69	-1.01	-0.37	.761
*VIQ*	-0.55	-0.87	-0.24	-0.54	-0.85	-0.23	.957
*PIQ*	-0.84	-1.20	-0.48	-0.80	-1.15	-0.44	.882
*VC*	-0.37	-0.66	-0.08	-0.46	-0.74	-0.18	.696
*PO*	-0.92	-1.24	-0.61	-0.76	-1.24	-0.61	.508
*PS*	-0.56	-0.99	-0.12	-0.56	-0.98	-0.13	1.00
*SEQ*	-1.13	-1.40	-0.87	-1.37	-1.63	-1.11	.255
*SIM*	-0.25	-0.58	0.08	-0.24	-0.56	0.08	.964
*SVAS*	-0.63	-1.01	-0.24	-0.68	-1.06	-0.30	.868
*SVAA*	-0.47	-0.95	0.11	-0.71	-1.19	-0.24	.524
*SAU*	-0.70	-1.10	-0.29	-0.72	-1.12	-0.32	.949
*IR*	0.07	-0.45	0.60	-0.59	-1.12	-0.08	.113
*DR*	-0.16	-0.64	0.33	-0.65	-1.12	-0.17	.205

Note: cognitive outcomes are means of z-scores corrected for the covariates age, the presence of comorbidities (ADHD and ASD), use of stimulant medication (MPH), educational status of patients, and family history of learning and behavioral problems. 95% CI = 95% Confidence Interval. FSIQ = Full-Scale Intelligence quotient, VIQ = Verbal Intelligence quotient, PIQ = Performance intelligence quotient, VC = Verbal Comprehension, PO = Perceptual Organization, PS = Processing speed, SEQ = Sequential processing, SIM = Simultaneous processing, SVAS = Sustained Visual Attention Speed, SVAA = Sustained Visual Attention Accuracy, SAU = Sustained Auditory Attention, IR = immediate recall, DR = delayed recall, DMD = Duchenne muscular dystrophy, NF1 = Neurofibromatosis type 1.

* p < .05 (two-sided)

#### Behavioral reports of parents and teachers

Outcomes of the behavioral reports of the DMD and NF1 group are displayed in Table 5.

**Table 5:** Behavioral reports of parents and teachers of the DMD and NF1 group.

Questionnaires with scales	DMD (n = 38)					NF1 (n = 38)					Definition of clinical range	*p*	*Effect size*
	Mean (SD)	Median	Min	Max	Clinical range (%)	Mean (SD)	Median	Min	Max	Clinical range (%)			
**CBCL**													
Anxiety/Depression	56.0 (6.6)	53	50	78	1 (2.6)	55.0 (7.9)	51	50	84	2 (5.3)	≥70	.120	
Withdrawn	60.5 (7.9)	60	50	82	4 (10.5)	59.9 (8.8)	58	50	88	4 (10.5)	≥70	.562	
Somatic complaints	58.3 (7.1)	57.3	50	76	3 (7.9)	58.3 (7.0)	57	50	72	2 (5.3)	≥70	.754	
Social problems	60.1 (6.9)	59.7	50	83	1 (2.6)	60.0 (7.1)	60	50	75	3 (7.9)	≥70	.925	
Thought problems	59.6 (7.5)	59.7	50	77	4 (10.5)	62.3 (7.5)	62.6	50	75	6 (15.8)	≥70	.096	
Attention problems	59.7 (6.2)	59	50	75	3 (7.9)	61.5 (6.3)	61	50	71	2 (5.3)	≥70	.155	
Rule-Breaking	56.7 (6.1)	55.5	50	71	1 (2.6)	55.2 (5.9)	53	50	73	2 (5.3)	≥70	.244	
Aggression	62.1 (9.3)	60.9	50	83	5 (13.2)	59.4 (7.7)	59	50	87	1 (2.6)	≥70	.256	
Intern. Prob.	57.2 (8.9)	57	34	75	9 (23.7)	55.1 (9.7)	53.2	41	78	7 (18.4)	≥63	.333^#^	0.3
Extern. Prob.	57.8 (11.0)	58.2	33	75	11 (28.9)	55.4 (10.0)	57.2	33	78	6 (15.8)	≥63	.335^#^	0.2
Total Prob.	59.9 (9.4)	60.2	41	77	12 (31.6)	58.8 (10.1)	59.3	34	78	11 (28.9)	≥63	.609^#^	0.1
**TRF**													
Anxiety/Depression	56.7 (5.1)	55.9	50	68	0	57.6 (5.9)	57.2	50	76	1 (2.6)	≥70	.460	
Withdrawn	58.2 (6.4)	57.3	50	77	2 (5.3)	58.6 (5.8)	57.2	50	81	1 (2.6)	≥70	.512	
Somatic complaints	52.4 (3.3)	50.2	50	62	0	53.5 (4.6)	51.2	50	67	0	≥70	.533	
Social problems	59.9 (4.8)	60.9	50	70	0	62.0 (7.8)	62	50	81	4 (10.5)	≥70	.240	
Thought problems	57.0 (5.8)	56.8	50	72	1 (2.6))	56.8 (6.8)	57	50	79	2 (5.3)	≥70	.740	
Attention problems	57.4 (6.1)	57	50	79	2 (5.3)	56.8 (5.5)	55.4	50	72	1 (2.6))	≥70	.621	
Rule-Breaking	54.7 (3.9)	54.9	50	68	0	54.8 (5.0)	53.7	50	68	0	≥70	.649	
Aggression	59.4 (5.7)	58.9	50	75	2 (5.3)	57.6 (6.1)	57.3	50	78	1 (2.6)	≥70	.156	
Intern. Prob.	55.9 (7.2)	56.1	38	71	5 (13.2)	58.0 (6.1)	57.6	45	75	6 (15.8)	≥63	.166^#^	-0.3
Extern. Prob.	57.1 (6.8)	56.6	41	73	5 (13.2)	55.0 (7.5)	55.5	41	74	6 (15.8)	≥63	.212^#^	0.3
Total Prob.	57.5 (6.0)	57	40	73	5 (13.2)	57.9 (6.2)	57.8	49	72	7 (18.4)	≥63	.776^#^	-0.1

Note: Mean scores (SD) are T-scores. CBCL = Child Behavior Checklist, TRF = Teacher Report Form, DMD = Duchenne muscular dystrophy, NF1 = Neurofibromatosis type 1, Inter. prob. = score of total internalizing problems, Extern. Prob. = score of total externalizing problems, Total prob. = total problems score. Differences between the DMD and NF1 group were assessed using Mann-Whitney U-tests, except for ^#^ which are analyzed using Independent sample t-test.

Parents of the DMD group reported that 23.7–28.9% of the males had internalizing or externalizing problems, whereas according to teachers 13.2% displayed internalizing and externalizing problems. Aggressive behavior was the most frequent observed behavioral problem in DMD (13.2%) according to parents (CBCL) responses. These five DMD males that displayed aggressive behavior were aged between 7,1–14,4 years. Problems with thinking and withdrawn were also reported by parents of the DMD group. Results further showed differences in the prevalence rates of behavioral problems reported by parents (CBCL) compared to those reported by teachers (TRF), with limited behavioral problems documented by teachers. Parents of the NF1 group, reported that 18.4–15.8% of the males had internalizing and externalizing problems, which is approximately comparable to the responses of teachers (15.8% internalizing and 15.8% externalizing). In particular, problems with thinking and withdrawn were documented by parents of the NF1 group, whereas teachers rated more social problems. Again, a difference in rates was found between parents (CBCL) and teachers (TRF) responses for the NF1 group. No significant differences were found between the DMD and the NF1 group on all subscales, the broadband scales internalizing- and externalizing problems and total problem scores (see Table 5).

A multivariate analysis was run to determine the effect of the covariates, age, comorbid diagnoses of patients (i.e. ADHD and ASD), use of stimulant medication (MPH), educational status of patients and family history of learning and behavioral problems on the CBCL and TRF broadband internalizing- and externalizing scales and the total problem scores. No differences were found between the groups on the internalizing CBCL scale, internalizing TRF scale score, externalizing CBCL and TRF scale scores and the total problem scores of the CBCL and TRF (see Table 6).

**Table 6:** Multivariate analyses of behavioral reports of parents and teachers of the DMD and NF1 group.

Questionnaires	DMD (n = 38)			NF1 (n = 38)			*p*
	Mean	95% CI		Mean	95% CI		
		Lower	Upper		Lower	Upper	
CBCL int.	58.3	54.8	61.9	54.4	51.0	57.8	.158
CBCL ext.	58.8	54.9	62.8	54.9	51.0	58.8	.210
CBCL total	60.8	57.3	64.3	58.2	54.8	61.6	.347
TRF int.	56.8	54.2	59.5	57.1	54.5	59.7	.900
TRF ext.	57.0	54.4	59.6	55.3	52.8	57.9	.416
TRF total	57.8	55.5	60.2	57.5	55.2	59.8	.864

Note: behavioral outcomes are means of t-scores corrected for the covariates age, the presence of comorbidities (ADHD and ASD), use of stimulant medication (MPH), educational status of patients, and family history of learning and behavioral problems. 95% CI = 95% Confidence Interval. CBCL int. = CBCL total internalizing problems scale score, CBCL ext. = CBCL total externalizing problems scale score, CBCL total = CBCL total problem score, TRF int. = TRF total internalizing problems scale score, TRF ext. = TRF total externalizing problems scale score, TRF total = TRF total problem score, DMD = Duchenne muscular dystrophy, NF1 = Neurofibromatosis type 1.

* p < .05 (two-sided)

## Discussion

Cognitive- and behavioral problems are well known comorbidities in the neurogenetic disorders, DMD and NF1. A lack of protein expression in the brain may be responsible for the development of these brain-related comorbidities in both disorders. Genotype-phenotype studies have investigated whether certain gene mutations result in specific and more severe phenotypes. In DMD, studies showed more severe cognitive and behavioral impairments in patients with mutations affecting the full-length and shorter brain isoforms, whereas in NF1 studies revealed more pronounced impairments in cognition, behavior and learning in patients with microdeletions. Since in both neurogenetic disorders, different proteins and regions are involved, we hypothesized that the cognitive and behavioral profiles of patients with DMD differ from patients with NF1. Results of reports of patient characteristics documented within the electronic patient files showed a statistical significant difference in frequencies of having an ADHD diagnose, with a higher prevalence rate for the NF1 group than DMD group. It is likely that the ADHD diagnosis is more difficult to establish in the DMD group due their physical immobility. Though, when exploring possible differences between the ambulant versus non-ambulant DMD group on the presence of an AD(H)D diagnosis we found no significant differences in our study sample.

In addition, surprisingly, no statistical significant differences were found between the groups on cognitive outcomes even after controlling for the covariates (age, comorbid diagnoses of patients (i.e. learning, neurodevelopmental, or behavioral disorders), use of stimulant medication (MPH), use of steroids, educational status of patients and family history of learning and behavioral problems). Results of reported behavioral problems by parents and teachers also displayed no significant differences between the DMD and NF1 group.

### Cognitive outcomes

The intelligence quotients of our total DMD group were in general in accordance with previous data, with an overall mean FSIQ that was approximately one standard deviation below the population mean [16]. No discrepancy between verbal IQ and performance IQ was found within our DMD group. This may likely be due to the large number of patients with distal mutations (55.3%) in our study of, which is known that they exhibit lower intellectual abilities in general [59]. Despite that our DMD group exhibit more difficulties on all intellectual tasks, their performances were not significantly lower compared to the NF1 group. However, the IQ distribution levels revealed that our DMD males predominantly fell within the low to low-average range, whereas the NF1 males performed low to normal. Higher rates of intellectual disability (FSIQ <70) have been described previously for DMD (30%) than for NF1 (4–8%), with most patients with NF1 falling in the low-average to normal range [16, 31, 59]. The IQ of our NF1 group was comparable to previous findings (IQ mean of 90) [4], which is on average comparable to the general population. Though, a variation in scores was noticed in our NF1 group, underscoring the heterogeneity in IQ in NF1 [33]. We found no correlation between the social-demographic status of the patients and their IQ levels.

Deficits in verbal span and working memory have long been documented as consistent cognitive features of DMD, but similar characteristics have been described for NF1 [15, 18, 33, 60–63]. Within the present study both groups equally displayed lower sequential processing outcomes that were likely independent of IQ. Both especially exhibit difficulties in recalling information that increase in load in sequential order. On delayed memory and recognition memory, both groups performed comparable and approximately normal. These findings emphasize that males with DMD often display a limited verbal short-term memory and span capacity in the recall of specific sequence information, but not in consolidation or retrieval [61]. Seeing the influence of limited verbal capacity on language development, attentional processes and learning it is important that the verbal memory problems are indicated at an early age. Particularly as it is shown that short-term memory and verbal span capacity are more powerful predictors for academic attainment of reading, writing and math than IQ [60–62, 64]. Furthermore, patients with delays in verbal span capacity seem not to grow out their deficit [62], underscoring that early diagnosis and treatment of cognitive and academic problems should be part of regular clinical care of both neurogenetic disorders [65, 66]. In terms of psychological interventions clinicians may address tools that enhance or stimulate the learning of verbal auditory information, such as remedial teaching at school [67]. Cognitive training for instance working memory training seems also a beneficial tool for children with short-term memory problems and learning disabilities [67, 68], and it efficacy for patients with DMD and NF1 with comorbid learning disabilities should be investigated in future studies.

With respect to processing speed and visuospatial abilities (simultaneous processing), we found comparable outcomes for both groups. In DMD, most studies reported normal visuospatial abilities, but for NF1 the visuospatial disabilities are known cognitive features [15, 31, 69, 70]. A possible explanation for the absence of visuospatial disabilities in our NF1 group may depend on the less sensitive cognitive tasks that we used in current study. For NF1 regular clinical care at CNL, patients underwent various visuospatial and visuomotor tests, however certain tests that are part of the NF1 protocol (i.e. Rey-Osterrieth Complex Figure test) are not collected for patients with DMD. This limited our possibility in comparing visuospatial outcomes in which patients with NF1 display great deficits [4]. Furthermore, in previous NF1 literature the visuospatial disabilities were found in groups that included males and females with NF1. In current study, we compared the cognitive profiles of a male DMD group and a male NF1 group. It is known that gender in NF1 strongly influences phenotype expression and it is suggested that the clinical heterogeneity in NF1 likely results from an interplay between genomic determinants such as gender and neurofibromin functioning [71]. This may explain why we did not found the visuospatial disabilties in our NF1 male group. It would be interesting to address the differences in phenotypes of NF1 male and females in future studies.

On sustained deficits in visual as well as auditory attention we found no differences between our groups. Within both neurogenetic disorders attention deficits are frequently reported, but to date most DMD and NF1 studies reference the prevalence rates of AD(H)D as a marker of presence of attention problems, with little to no use of direct neurocognitive measures of attention. Only three previous DMD study used a cognitive attention task to estimate attention [15, 18, 72], whereas two other studies used a processing speed task or verbal memory task [69, 70]. Studies addressing attention deficits solely based on ADHD prevalence rates should be interpreted with caution, because evidence is growing on the distinction between patients with ADHD with predominantly behavioural features (hyperactive/impulsive) and patients with the cognitive phenotype (inattention) [33]. Each type is suggested to have its own type of impairments, developmental trajectories and underlying neurobiology, which requires differentiation in diagnosis as well as in treatment [33].

We noticed that 55.3% of our DMD group, had distal mutations abolishing the production of *Dp140* and it is suggested that these males have more severe cognitive impairments [23, 24, 26]. Additional post-hoc analyses checked whether the DMD males with mutations affecting *Dp140* production (*Dp140-*, N = 10), DMD males with intact *Dp140* (*Dp140+*, N = 21) and males with NF1 (N = 38) differed. After applying Bonferroni correction we found a trend (*p* = .60) for the group *Dp140-* indicating that these patients performed less well on processing speed compared to the other two groups (*Dp140+* and NF1). It may be considered that in neurogenetic disorders not all cognitive functions are fully attributable to the genotype, but environmental and perinatal factors including maternal factors (e.g. stress, malnutrition, hypertension, substance (abuse) and fatal factors (hypoxia, low birth weight, prematurity) may be contributable and determinative for the phenotypes of patients as well [73].

### Behavioral outcomes

On average, males of both groups fell below the clinical cut off values on all syndrome scales, the broadband scales and the total scale scores of the CBCL and the TRF, representing that parents and teachers reported no significant elevated behavioral problems. More detailed analyses on abnormal ranges of the groups showed that parents and teachers of males with DMD more often reported aggressive behavioral problems. Prednisone is the standard prescription to stabilize muscle strength, extend ambulation and stand abilities in DMD and it is known that boys who take steroids exhibit more externalizing behavioral problems i.e. aggressive behavior than boys taking no steroids. This may explain the higher rates of aggressive behavior in our DMD group. However, results on the relation steroid use and higher incidences of externalizing behavioral problems are equivocal [58, 74, 75]. Furthermore, patients with DMD deal with physical milestones during the disease course, which may induce aggressive behavior as well. For instance, our males were aged between 7–14 years and this is the age-range at which patients with DMD are confronted with loss of ambulation.

Within the NF1 group, parents more often reported difficulties in thinking and withdrawn, whereas teachers often reported social problems. It is interesting that the behavioral problems reported by parents and teachers of both groups differed in rates, with higher incidences reported by parents. In DMD, it is known that parent ratings are higher probably due to the parents perception of the magnitude of problems belonging to the illness and the increased parental stress resulting from difficult parent-child interactions [76, 77]. Our findings emphasize that screening of behavioral problems should be done by evaluating different perspectives (i.e. parents, teachers, patients and clinicians) on patients functioning [78]. This is particularly important in neurogenetic disorders due to the presence of more than one cognitive or behavioral comorbidity and their overlap in symptoms. Furthermore, the CBCL may be no suitable instrument for screening behavioral problems, which we previously described in our systematic review [78]. For clinicians it is important to know that results of the CBCL should be interpreted with caution and no definite diagnoses should be made solely on the basis of this instrument, as the symptom items of the CBCL subscales have no conceptual link with diagnostic criteria of behavioral disorders [79]. In addition, insufficient psychometric properties have been found recently for the CBCL especially for patients with DMD [78]. This may explain why some of the anomalies such as the lack of social problems in our DMD sample are not found, while 25% of them had a diagnosis of ASD. Many years the CBCL was used as gold standard in our Centre. However, the diagnostic work-up of patients with DMD is currently adapted due to our recent sensitivity findings of the CBCL [78].

### Neurophysiology in relation to cognition and behavior in DMD and NF1

In both neurogenetic disorders, the affected proteins (i.e. dystrophin in DMD and neurofibromin in NF1) are expressed in a wide variety of nervous tissues including neurons and glial cells (e.g. astrocytes, oligodendrocytes) in the brain [7, 10, 30, 80–82]. A loss of the affected proteins result in functional and structural alterations of neurons and glial cells particularly located in corticostratial circuits and the hippocampus [7, 30, 80, 81, 83]. For instance, by interacting with other components of the dystrophin-glycoprotein complex (*DGC*), such as syntrophin, the brain variant of the full-length dystrophin protein isoform (*Dp427*_*B*_) links to inhibitory γ-aminobutyric acid type A (GABA_A_) receptors at the postsynaptic neural membrane [7, 84]. A lack of dystrophin results in a decreased density of GABA_A_ receptor clustering of receptor subunits at inhibitory synapses [83, 84]. Aberrant anchoring of GABA_A_ receptors causes an increased extrasynaptic expression of GABA_A_ receptors, which triggers a disruption of calcium homeostasis and makes cells vulnerable to necrosis [85, 86]. Dystrophin deficiency also induces altered excitatory synapse functions and organizations i.e. abnormal enhanced NMDA receptor activation [10]. In NF1, the decreased production of neurofibromin causes reduced Ras signaling molecule, leading to increased GABAenergic inhibition in the hippocampus due to impaired long-term potentiation [30, 81]. Furthermore, neurofibromin is localized at excitatory synapses postsynaptically where it interacts with the NMDA receptor [30, 81]. Overall, in both disorders neuronal alterations in GABA_A_ and glutamate functions are found and these have been linked to the presence of neurocognitive deficits [7, 10, 30, 80–82]. It is tempting to speculate that due to the comparable neuronal defects we found no differences between the DMD and NF1 group. Though in DMD an increased excitation is described whereas in NF1 an increased inhibition is found, respectively. Further research should elucidate whether this dissimilarity effects the presence and severity of cognitive deficits. Another possible etiology for the cognitive (and also learning) abnormalities in DMD and NF1 are glial dysfunctions (e.g. astrocyte abnormalities), but their contributory role needs further investigation [7, 30]. The proposed mechanisms that may underlie the cognitive phenotype in DMD are versatile. In addition, neuroimaging studies revealed individual variability in brain structures, networks, perfusion and metabolism [87]. Future studies should link the cognitive outcomes to genetics (i.e. dystrophin isoform expression and neurophysiology) and neuroimaging, to better determine the factors involved in the presence and severity of the DMD cognitive phenotype [87].

### Limitations and future perspectives

This study has some limitations. At first, not all collected data of the groups could be evaluated as other standard protocols have been used for regular clinical care for DMD and NF1 in our outpatient clinic. Therefore, academic skills for instance reading, writing and math that are often impaired in both groups were not assessed in current study. The available data of the cognitive test used were adequate for the purpose of current study. Since these measures evaluate whether the frequent observed cognitive problems (i.e. deficits in intellectual abilities, verbal (working) memory and attention) that are found in both neurogenetic disorders are also shown in current study. The data of behavioral outcomes of the CBCL which is the gold standard for evaluating the presence of frequent observed comorbidities such as ADHD and ASD may lack some sensitivity in our groups. This is further described within the sixth limitation below.

Secondly, due to the differences in neuropsychological batteries certain data were missing for which we applied stochastic regression imputation, but this does not take patients physical abilities into account. Thirdly, all participants of the present study were referred to the outpatient clinic CNL, and these patients frequently have more (severe) learning, cognitive or behavioral than other patients with DMD or NF1, making our results likely less generalizable. Although, most prevalence rates of comorbid neurodevelopmental diagnoses and certain cognitive outcomes were in line with previous literature on cognition in patients with DMD and NF1. Furthermore, the group with DMD having these comorbid cognitive and behavioral diagnosis are limited. This makes it difficult to have a required minimum sample size based on power analysis for finding statistical significant differences between the DMD group and any other neurogenetic or dystrophy disorders. However, the additional power analysis displayed a power of 20% based on the sample size of current study. Fourthly, we solely included participants aged 6–16 years to allow for the administration of the cognitive test and behavioral questionnaires, standardized for the Dutch population which also limited our study sample group. However, cognitive and behavioral functions undergo major changes throughout childhood development, making mean group comparisons with large distributions of performances of young children (i.e. aged 6–7) and older children (15–16 years) difficult. Fifthly, current study used both the WISC-III and KABC-II. Additional correlations showed only small correlations between the subtests of the batteries indicating that in this study the subtests measured different domains of cognition. In DMD time restricted WISC tasks may negatively influence the outcomes due to less upper extremity functioning, therefore we choose to administer both batteries. Sixthly, a disadvantage of using retrospective data was that the reported comorbid diagnosis differed from data collected by measurements completed by parents and teachers. For instance 23% of DMD patients had a diagnosis of ASD but the parent and teacher rating did not identified significant social and behavioral concerns. Additionally, when comparing the reports on ADHD diagnoses we found a significant difference between NF1 and DMD, with a higher prevalence rate for the NF1 group. It is likely that due to the lack of sensitivity of the CBCL measure we did not objectively found a significant difference between the groups on ASD and ADHD. Future prospective studies should include more sensitive measures such as a clinical structured interview, the Autism Diagnostic Observation Scale [22]. Nevertheless, our findings emphasize that comorbidities in cognition, behavior and learning difficulties may arise in both neurogenetic disorders. Early cognitive and behavioral (re)evaluations are required and should be part of standards of care, in order to facilitate treatment as early as possible when necessary. Future longitudinal studies in DMD and NF1 should evaluate whether patients future grow in or out of their cognitive, behavioral and learning comorbidities. A nice addition to the NF1 literature would be to evaluate genotypes-phenotypes in severity and impact of cognitive, behavioral and learning comorbidities. These analyses could not be carried out in current study, since none of the NF1 patients of which mutation information was available (n = 32) had microdeletions.

It can be speculated that we found no differences in cognitive profiles since not only genetic mutations are responsible for the neurocognitive outcomes, or it is likely that the cognitive and behavioral tests used lack sensitivity for these specific neurogenetic disorders. Although, when the profiles are associated with mutations we would aspect to find differences during diagnostics or during interventions. Recently for instance, positive effects have been observed when using a general cognitive intervention i.e. computerized working memory training in patients with DMD and a comorbid learning disability and these effects seem similar to those found in patients with learning disabilities without DMD [88]. This suggests that also general interventions are applicable for patients with neurogenetic disorders despite that the comorbidities may be associated to the genetic mutations. It can be wondered which other factors than mutations are responsible for the cognitive and behavioral profiles of patients with neurogenetic disorders. There is growing evidence that epigenetic factors (e.g. maternal stress) may modulate brain development as well [73].

## Conclusion

The cognitive features of patients with DMD considerably overlap with those of male patients with NF1. It suggests that brain-related comorbidities in cognition are not only caused by gene mutations resulting in a lack of one specific protein, but also depend on other protein interactions and on neuronal and glial functional and structural alterations. However, some differences in clinical features were noticed between the DMD and NF1 group, for instance the IQ levels of the DMD group were more distributed to the left compared to the NF1 group. Furthermore, the parental reported ADHD prevalence rate was higher within the NF1 group compared to the DMD group. With regard to other behavioral features, aggressive behavior was more often reported by parents and teachers of the DMD group, whereas in NF1 parents and teachers frequently reported problems with thinking, withdrawn and social behavior. Clinicians should keep in mind that in both disorders one or more comorbidities may occur, that symptoms may overlap and that the severity of symptoms may variate between patients. This underscores that (re) evaluations and monitoring of cognitive development and behavioral functioning is required in both neurogenetic disorders. Possible cognitive or behavioral implications for treatment in both disorders could be for instance remedial teaching, cognitive (working memory) training, social training, psycho-education for patients, parents and teachers and neuropsychopharmacology.

## Supporting information

S1 Table Mutations of the NF1 patients. (DOCX)
