# Supplementary material for: Cognitive and behavioral functioning in two neurogenetic disorders; how different are these aspects in Duchenne muscular dystrophy and Neurofibromatosis type 1?
Source: PLoS One. 2022 Oct 10;17(10):e0275803. doi: 10.1371/journal.pone.0275803 (PMC9551631; doi:10.1371/journal.pone.0275803)
Supplement: S1 Table — (DOCX) [file pone.0275803.s001.docx]

| **Patient** | **Genetic mutation** |
| --- | --- |
| 1 | Spontaneous mutation r2616 x |
| 2 | Mutation c3047-3048delGT |
| 3 | Spontaneous mutation 6841delC (ex37) de novo |
| 4 | Mutation L549P |
| 5 | Missing |
| 6 | Spontaneous mutation 1855del7 |
| 7 | Spontaneous mutation R816X |
| 8 | Mutation c.6949_6951 deletion |
| 9 | Mutation c.205-1G>T |
| 10 | Clinical diagnosis not genetically confirmed |
| 11 | Deletion exon 2 and 3 NF1-gen |
| 12 | Mutation c.5758_5762del5, p.Leu1920fs deletion |
| 13 | Missing |
| 14 | Spontaneous mutation 2326-2A>T |
| 15 | Missing |
| 16 | Familial mutation 4368-2A>C |
| 17 | Mutation w571x |
| 18 | Spontaneous mutation G543X |
| 19 | Genetically confirmed, mutation missing |
| 20 | Mutation 1260+3A>C in exon 9 |
| 21 | Deletion exon 28 |
| 22 | Spontaneous mutation 4006delC |
| 23 | Mutation L2104R |
| 24 | Clinical diagnosis not genetically confirmed |
| 25 | Mutation 4117delA |
| 26 | Mutation L695P, 2084T>C |
| 27 | Missing |
| 28 | Familial mutation 24-27b (c.4110+275_4772+25534dup41480) |
| 29 | Deletion exon 47, P2696P, 8088G>A |
| 30 | Familial mutation R26'16X,7846C>T |
| 31 | Familial mutation c.2850delGinsTTAC, p.Gln950fs |
| 32 | Spontaneous mutation 477delGAGinsAGA |
| 33 | Pathogenic mutation 3870+1G>C |
| 34 | Missense mutation p.Arg1809Ser |
| 35 | Familial mutation 1527+5G>T |
| 36 | Pathogenic mutation exon 41 7389delC |
| 37 | UV L995P |
| 38 | Pathogenic mutation 246delTC |

**Supplementary Table 1. Mutations of the NF1 patients**

Note: Genetic mutations of the N=38 males with Neurofibromatosis type 1. Of n=2 clinical diagnosis not genetically confirmed, n=1 genetically confirmed but mutation information is missing, n=4 missing.
